# The 2011 *Retrovirology Prize *winner Masao Matsuoka: forward looking and antisense

**DOI:** 10.1186/1742-4690-8-102

**Published:** 2011-12-15

**Authors:** Kuan-Teh Jeang

**Affiliations:** 1 Laboratory of Molecular Microbiology, National Institute of Allergy and Infectious Diseases, the National Institutes of Health, 9000 Rockville Pike, Bethesda 20892, MD, USA

## Abstract

Masao Matsuoka wins the 2011 *Retrovirology Prize*.

## 

Seven years ago, *Retrovirology *inaugurated an annual prize to recognize the achievements of a deserving retrovirologist [1]. The *Prize *was founded by the Ming K. Jeang Foundation, a philanthropic charity based in Houston, Texas, which has provided scholarships at Houston schools, the University of Arizona, and the Johns Hopkins University School of Medicine. Previous winners of the *Retrovirology Prize *include Stephen Goff, Joseph Sodroski, Karen Beemon, Ben Berkout, Thierry Heidmann [2], and Michael Malim [3]. Annually, the *Retrovirology Prize *recognizes an outstanding mid-career scientist who is at the height of his/her scientific productivity and who is expected to have many future years of high achievement. In even numbered years, the Prize is awarded to an HIV virologist, in odd numbered years it is for a non-HIV retrovirologist.

For 2011, the Editors of *Retrovirology *have selected Masao Matsuoka as the recipient of the *Retrovirology Prize *(Figure 1). Professor Masao Matsuoka is a physician by training and an oncologist by medical specialty. He graduated from Kumamoto University School of Medicine and did a postdoctoral fellowship at the University of California Berkeley before returning to Japan where he currently is the Professor and Director of the Institute for Virus Research at Kyoto University. For the past 25 years, Matsuoka has been a pioneer and a leading figure in human T-cell leukemia virus type 1 (HTLV-1) research. He has made many important discoveries with two particularly notable findings. HTLV-1 infection frequently becomes latent, and one of the major puzzles in the field is how this occurs. Matsuoka's seminal contribution here is his discovery that the 5' LTR DNA sequence of the HTLV-1 provirus is preferentially methylated while the 3' LTR of the provirus is not. His work in this area is independent of and contemporaneous with similar observations made in human immunodeficiency virus type 1 (HIV-1) research and has added to our insights on how retroviral latency is achieved and how it might be thwarted by employing targeted therapeutic agents.

**Figure 1:**
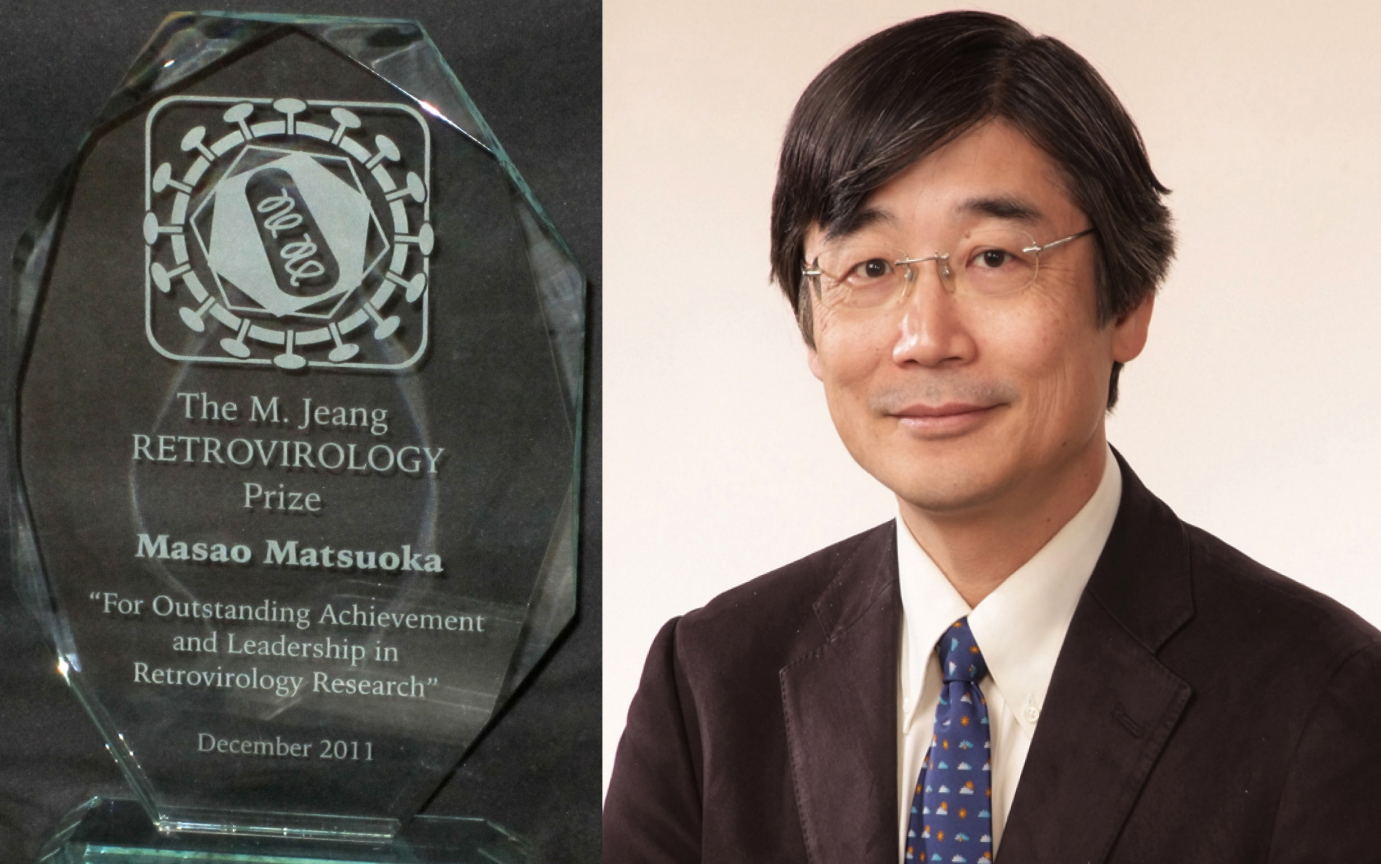
**A picture of the *Retrovirology *Prize trophy (left) and Masao Matsuoka (right)**.

Because Matsuoka found that the 3' LTR of the HTLV-1 provirus is surprisingly hypomethylated, this led him to consider whether such a state would favor an antisense transcript originating from this end of the proviral genome. HIV-1 researchers have long ago discounted the importance of a 3' LTR driven antisense transcript. Yet, in the last decade, independent results from Mesnard (France) and Matsuoka (Japan) have strongly established the existence and biological importance of an HTLV-1 antisense transcript called HBZ. The HBZ RNA encoded by the minus strand of the provirus is capable of encoding a bZIP protein, and Matsuoka and his colleagues have shown that this RNA and its protein are expressed in all Adult T-cell leukemias (ATLs). Importantly, Matsuoka is the first to demonstrate that the expression of HBZ promotes the proliferation of T cells, and he has reported that HBZ expressing transgenic mice develop T cell lymphomas.

Professor Matsuoka has been a founding member of *Retrovirology's *editorial board. To understand better Masao's views, I asked for his answers to several questions.

**KTJ: **Tell me what you find the most rewarding about being a retrovirologist?

**MM: **Human retroviruses are big threats to humans. They evolve to replicate elaborately in human cells using their small genomes. Whenever I find clever mechanisms of retroviruses, they never failed to surprise me. At the same time, I feel the challenge to eliminate them from humans and to prevent retroviral diseases. As a retrovirologist, I am fascinated to study these small, but immensely important genomes with their ingenious mechanisms.

**KTJ: **Did you ever have second thoughts about being a scientist? What would you have done if you did not become a scientist?

**MM: **When I was a boy, I was fascinated with living fish and insects. I was very much interested in living animals. It is a reason that I love fishing. If I did not become a scientist, I would have liked to be a fisherman. However, for Japanese fishermen, it is very competitive to catch good fish for "Sushi". I am skeptical that I can succeed as a fisherman. I guess that being a scientist is better for me.

**KTJ: **The 21^st ^century is said by many to be the "Pacific century" --- how do you see science being different in the 21^st ^century compared to the 20^th ^century?

**MM: **I am confident in the 21^st ^century that people in the Pacific countries will be influential in science. A striking feature of this region is diversity in many aspects, including race, culture, politics, and so on. I hope that this huge diversity generates new science, and contributes to human society to solve many challenging puzzles like climate changes, and ever changing diseases (diabetes mellitus, cancer, infectious diseases, etc.), and the exhaustion of natural resources. Of course, none of these issues are easy to be solved. I really hope that diverse people in Pacific countries contribute to these tough problems.

**KTJ: **Young scientists face many obstacles today in developing their careers --- what do you see as some obstacles facing young Japanese scientists and how would you advise them?

**MM: **In most Japanese universities, associate and assistant professors work under the supervision of full professors. Full professors get grant money from the government or funding agencies, and they usually determine the research theme for young scientists. It is not an actual independent position. I would like to recommend young scientists to be independent as possible as they can or to choose a good boss who permits them to do independent research.

**KTJ: **Someone told me once that every 10 years he reinvents himself. What do you see yourself doing 10 years from now that might be different from what you are doing today?

**MM: **I agree that reinventing oneself is a nice and cool idea. However, I think that people (except rare persons) tend to behave as they always are. Before I moved to this Institute, I was a clinician, and took care of many patients in the hospital. I spent small efforts for my research at that time (half clinician, and half scientist). However, now I spend much more time for my research in Institute for Virus Research, Kyoto University. My past background is good experience for me since I can appreciate different points of view for retrovirology. Actually, I think understanding how the virus induces various clinical conditions (inflammation, hypercalcemia, immunodeficiency, etc.) from virological and clinical points of view provide informed and synergistic perspectives. Sometimes, these different views lead to new ideas.

**KTJ: **You have published 6 papers [4-9] in *Retrovirology *--- tell me what do you think about Open Access --- should this principle be important to Japanese scientists, why or why not?

**MM: **I strongly agree with *Open Access*. Access to knowledge should be open to anyone who wants it. Recently, Japanese government and funding agencies do not request open access to scientists. This policy should be changed. Kyoto University has its own repository (Kyoto University Research Information Repository), Kurenai (that means "truly red" in Japanese), to access publications from Kyoto University. The world is sometimes unfair. But, science should be open and fair for anyone who wants to know new findings and knowledge. It should be the concrete basis for science.

**KTJ: **When the time comes and you retire from doing science, how and for what would you like to be remembered?

**MM: **When I retire from science, I would like to be remembered as a researcher who connected clinical science and retrovirology. Of course, I also study drug development for HIV-1 and resistance mechanisms. I am confident that there are many other important contributors to this field of HIV.

**KTJ: **Finally, if you have an opportunity to address a gathering of all the world leaders at the United Nation, in 250 words or less --- what would you say to them?

MM: Now, the world has become closely connected more than at any other time of human history in many aspects. Economies are tightly linked, and an economical crisis like the Greek crisis in one country quickly affects many other world economies. Climate changes also influence the world. In the latter, a limitless desire of human exploitation of our environment has exacerbated these situations. A new viral infection quickly spreads within weeks like SARS. The HIV pandemic is a big tragedy to human, and avian influenza will be a new threat. To respond to these infections, we need a new platform of collaboration and friendship. Thus, we must appreciate that people and countries are indivisibly united. However, the sad thing is that people of the world are stuck in old thinking. We need to understand that in today's world it is not possible for one country to pursue mainly its own benefit.

The "chyu-you (derived from the famous Chinese book)" means the balanced state or person with harmonized character, which is appreciated by people for a long time in Japan. I hope that people and countries should keep this word in mind, and exercise control over themselve. The host and the pathogen co-evolve to adapt to each other. It is time for humans to harmonize the world and society. Science is an important arena which gives us many opportunities to achieve this.
